# The Role of Artificial Intelligence in Orthodontics for Determining Skeletal Age Based on Cervical Vertebra Maturation Degree: A Comprehensive Review

**DOI:** 10.1002/hsr2.71487

**Published:** 2025-11-10

**Authors:** Pegah Farzanegan, Mobina Zarabadi, Shaghayegh Najary, Mohammad Ali Tahmasbi, Mohammad Behnaz

**Affiliations:** ^1^ Student Research Committee Babol University of Medical Sciences Babol Iran; ^2^ Network of Interdisciplinarity in Neonates and Infants (NINI) Universal Scientific Education and Research Network (USERN) Tehran Iran; ^3^ Department of Pediatric Dentistry, School of Dentistry Zanjan University of Medical Sciences Zanjan Iran; ^4^ School of Dentistry Shahid Beheshti University of Medical Sciences Tehran Iran; ^5^ USERN Office Shahid Beheshti University of Medical Sciences Tehran Iran; ^6^ School of Dentistry Tehran University of Medical Sciences Tehran Iran; ^7^ Master of Science in Communication Systems, School of Electrical Engineering Sharif University of Technology Tehran Iran; ^8^ Dental Research Center, Research Institute of Dental Sciences, School of Dentistry Shahid Beheshti University of Medical Sciences Tehran Iran

**Keywords:** Artificial intelligence, cervical vertebra, orthodontics, skeletal age

## Abstract

**Background and Aims:**

Dentofacial orthopedic treatment planning highly depends on the estimation of skeletal growth peak. In most cases, chronological age and biological age differ, some techniques estimate skeletal age by analyzing cervical vertebrae maturation (CVM) staging on lateral cephalograms. In this study, we aimed to review the different applications of AI in orthodontics and specifically discussed the different designs of AI models used for CVM estimation.

**Methods:**

Comprehensive searches was conducted across databases including PubMed, Web of Science, Google scholar, Embase, and Scopus using keywords such as orthodontics, cervical vertebra maturation, skeletal age and artificial intelligence.

**Results:**

Utilizing AI algorithms in assessing CVM‐based skeletal age enhanced the accuracy of diagnosis, reduced analysis time and minimized observer variability. Deep learning techniques, especially convolutional neural networks (CNNs), have demonstrated promising results in identifying CVM stages from lateral cephalometric radiographs.

**Conclusion:**

AI algorithms can assist orthodontists in identifying CVM stages on lateral cephalograms. While the accuracy of AI algorithm depends on factors such as data set size, labeling methods, and model design, expert supervision and a solid understanding of CVM principles remain essential. AI should be considered as a supportive tool, not a replacement for clinical judgment.

## 1 Introduction

Estimation of skeletal growth peak affects the treatment planning process in the field of dentofacial orthopedics [1]. Patients who received functional orthodontic appliances before reaching skeletal maturation showed superior results in achieving a harmonious relationship between the upper and lower jaws [2]. There exists a discrepancy between chronological age and biological age in most cases; therefore, some methods are currently applied for the estimation of skeletal age based on assessing the developmental stages of hand and wrist bones as well as cervical vertebrae maturation (CVM) [3, 4]. Lamparski was the first to propose a method for evaluating the maturity of the cervical vertebrae using lateral cephalometric radiographs [5]. This approach consisted of evaluating the morphological changes in the three specific cervical vertebrae (C2, C3, and C4) as indicators of skeletal development. The approaches and standards suggested by Lamparski on CVMS have been improved and revised several times throughout time [4, 6]. The classification system established by Baccetti, Franchi, and McNamara demonstrated a significant degree of accuracy and adherence to the hand and wrist method (HWM) [7, 8]. However, it may be challenging and time‐consuming for dentists and orthodontists to classify the stages of maturation for cervical vertebrae. A meta‐analysis by Cericato et al. evaluated the accuracy of CVM method for the assessment of skeletal maturation and the results revealed a significant correlation between CVM method and pubertal growth spurt, and higher correlation was seen for female patients than for males. Therefore, CVM assessment is a reliable method to replace Hand‐wrist radiographs [9].

Throughout the years, several technologies have been identified in dentistry practice. Artificial intelligence (AI) is an emerging technological development that has shown significant potential in offering more accurate diagnosis and faster treatment planning [10]. Machine learning (ML) is a widely used type of AI that enables computers to be trained and acquire knowledge through analyzing data and observations without the need for intricate programming [11]. The accuracy of the findings obtained by the system would increase in proportion to the number of primary data provided for training. Deep learning (DL), which is categorized under the general heading of machine learning, exhibits enhanced adaptability and has the ability to extract abstract characteristics from unprocessed data [12]. Machine‐learning models with multiple processing layers are used in the field of medical imaging to evaluate radiographs with the purpose of identifying anatomical landmarks and detecting anomalies. Deep learning, using convolutional neural networks (CNNs) has also been demonstrated to have remarkable potential for dental image diagnostics [13].

CVM analysis consists of a subjective evaluation with low reproducibility [14, 15, 16]. Investigations have demonstrated that the application of AI will result in more precise and reproducible estimations [17, 18]. There are several factors that could have an effect on the final results on the accuracy and precision of the AI models including the number of datasets used for model training, manual or automated landmark identification, standardization of data with inter‐ and intra‐observer agreement, etc [19].

In this literature review, we proposed a brief overview of AI applications in the field of orthodontics. We emphasized the different AI models used for skeletal age estimation based on cervical vertebra maturation and discussed the different methods and models suggested by different studies.

## 2 Types of AI Models Used for Determining Growth Pattern

### 2.1 Machine Learning

Machine learning (ML) is defined as a subfield of artificial intelligence and a study of algorithms and statistical models for processing tasks without the necessity of prior rules and knowledge [20]. ML can be used in many fields, from diagnosis to treatment planning in different disorders. ML in orthodontics can be used for various purposes including determination of skeletal classification, duration of orthodontic treatment, the need for orthognathic surgery and providing an accurate treatment plan [8]. Recent years the use of ML has been widespread in many medical fields such as dentistry [21]. In ML, a feature vector is used as a describing element. The process of feature makes a data set from raw data by extracting the features [22]. Four different approaches can be utilized for algorithm models. In supervised learning, experts label the training data set to be learned by the ML algorithm to make a model. In this manner, the final model is created by using key features of the labeled training data set as input which makes an output in the form of either continuous or discrete variable, called a regression or classification model respectively. In unsupervised learning, a model is created by unlabeled data set and the algorithm that transforms features to another vector to solve the problem. Two other models are reinforcement learning and semi‐supervised learning [22, 23, 24]. We can benefit from application of ML techniques in various fields of dentistry, including disease diagnosis, assessment of prognosis and treatment planning. Using a proper technique needs analyzing of the problem accurately [21, 25, 26].

### 2.2 Deep Learning

In recent years, the use of deep learning (DL), a subset of ML, has increased remarkably. DL imitates human intelligence in complex ways to discover features in unstructured datasets [27, 28]. DL recruits various layers of nonlinear units to obtain favorable information and knowledge from great amounts of data. The obtained data is then utilized to make predictive results [25]. DL enhances medical‐related procedures including more effective treatment plans, making clinical decisions and image interpretations [29, 30]. In addition, DL has a promising role in the field of orthodontics, including identifying the necessity of orthognathic surgery, lateral cephalometry landmark detection and skeletal classification [31, 32]. DL algorithms mainly work based on complex neural networks in different ways, leading to various methods as explained in the following.

### 2.3 Artificial Neural Network

Artificial neural network (ANN) defines as a mathematical model based on the signals of the human brain [33]. Artificial neurons are the basic units of ANN. ANN consists of input and output values as vectors. Input layer is the first layer of ANN. Between both input and output layers, different numbers of hidden layers can be designed. Various numbers of artificial neurons are made within hidden layers. Within artificial neurons, linear or nonlinear input values can be modified through different activation functions and the output transmits to the input of the next layer [34]. ANN models can be beneficial in many fields of medical research including image processing, processing complicated problems and classification [19, 33, 35].

### 2.4 Convolutional Neural Network

Convolutional neural network (CNN) is considered as a sort of deep learning neural network. It has a notable performance in image classification and recognition [13, 34]. Within convolutional layers, multiple feature maps are made from the extraction of different patterns of the image. Pooling process works in reducing the computation and the size of the image. After mentioned process, the output connects with other connected layers to categorize the image. Applying CNN as an assisting tool has shown satisfying results in medical fields including radiology and dermatology [36, 37]. while in the field of orthodontics, it is making progress gradually. Makerem et al. proposed using a customized CNN model to determine the degree of cervical vertebral maturation by cephalograms. The CNN model had an accuracy of 95% in determining maturation degree of CVM and can be utilized more often in this field [25, 38].

### 2.5 Recurrent Neural Network

Recurrent neural network (RNN), in spite of the feed‐forward process in ANN, is based on saving the output of a layer to be fed as input to the next step. Such a process makes this category of neural networks an appropriate algorithm to handle sequential data, resulting in a dynamic behavior of the model. In the field of dentistry, there are such procedures as orthodontic facial recognition can benefit from this feed‐back principle in the processing of a sequence of data. Murata et al. proposed a diagnostic model employing RNN to objectively assess the patient's facial features for orthodontic treatment. RNN has been helpful in capturing the long‐term dependencies in data and, as a result, has outperformed the CNN model in terms of assessment accuracy [37].

## 3 Application of AI in Radiology

### 3.1 History of AI in Radiology

Artificial intelligence (AI) has been a hot topic in radiology for decades. In the 1960s, the first efforts were made to enter computer‐based systems in radiology [39]. In the 1980s, With the progress in technology, AI‐powered systems moved forward for automated segmentation [40, 41]. In the 1990s, digital radiography was introduced in the field of dentistry for periodontal disease, bone lesions, caries diagnosis, root resorption as well as cephalometric and craniofacial growth analysis [42, 43, 44, 45, 46]. At that time, a new digital subtraction method for tracing differences in radiography was introduced. It was used for condylar position changes or craniofacial growth by taking two images in the same geometry but different periods and then superimposed images were evaluated. Identical regions were equalized in contrast; therefore, the remaining changes were visualized, although the results were not accurate enough [4]. However, with the entrance of machine learning and CNNs in cephalometry, craniofacial growth was evaluated more accurately and faster [47].

### 3.2 Image Processing

As we know, medical imaging has a significant role in diagnosis and treatment, and it has been facilitated by image processing in the last two decades [48]. Image processing mimics the human visual system by evaluating image pixels. For each pixel and neighboring pixels, a feature vector is created and contrast, uniformity, colors, and edges will be determined [49, 50]. For example, the average color of all pixels is evaluated and then other pixels are compared to the average [49]. Segmentation methods can be classified into five groups [50]:

- Region Based.
- Edge Based.
- Threshold.
- Feature Based Clustering.
- Model Based.

### 3.3 Cephalometric Landmark Detection

The most crucial part of image processing is segmentation and the main purpose is to eliminate extra information for an easier processing and to divide radiographs into meaningful areas. Various features including changes in color or texture are converted to a vector for segmentation and the edges are identified [50, 51]. Automated segmentation enables fast and accurate identification with minimum errors compared to human evaluation. it can be used for the detection and identification of anatomical landmarks, even diseases, and abnormalities [52].

Cephalometry is an important radiography for determining craniofacial skeletal relationships, both qualitatively and quantitatively through angular and linear measurements and landmark detection [53]. Manual evaluation of cephalometric radiographs is rather time‐consuming even for experts. It was found that time spent on skeletal age assessment was decreased from 54.29 s to 35 s when using AI algorithms rather than manual methods, and the accuracy of assessment was increased from 94% to 99% by utilizing AI [54]. Thanks to AI‐powered systems, cephalometric analysis is now faster and more accurate. First, landmarks should be traced and then angles are measured by software such as Planmeca Romexis or Automatic Cephalon‐Diagnostic Solutions (ACDS) [55]. For landmark identification, there are various approaches including:

1. Image filtering and knowledge‐based landmark search.
2. Model‐based approach.
3. Soft computing or learning approach.
4. Hybrid approach [55].

With the application of AI in cephalometric analysis, accuracy was still a question. Systematic reviews concluded that AI performed accurately in cephalometric analysis and has shown the same or even higher success rates compared to manual analysis [53].

## 4 Application of AI in Orthodontics

### 4.1 Segmentation and Landmark Identification

Imaging evaluations are an inevitable part of orthodontic diagnosis, treatment planning, and follow‐ups. Since the time it was first introduced in 1931 by Broadbent, lateral cephalometric analysis has continued to be the primary diagnostic technique used in orthodontics [56]. This analysis is crucial in treatment planning as it involves identifying specific anatomical points on X‐ray radiographies and then measuring various distances, angles, and ratios to provide clarity on the craniofacial structures. Due to the complexity of the skull structure, which is projected in a two‐dimensional form on a cephalogram, the images often display overlapping characteristics. Moreover, factors such as anatomical differences among individuals, variations in head positioning during image capture and facial asymmetry make it difficult to locate landmarks on lateral cephalograms. This manual process is time‐consuming and leads to inconsistencies between clinicians and even within the same clinician [57, 58]. Since inaccurate cephalometric analysis can have serious repercussions, there is a need for a self‐adjusting algorithm that can accurately detect cephalometric landmarks [59]. In the field of orthodontics, both photography and radiography are required including lateral cephalometric and panoramic radiography as well as cone beam computed tomography (CBCT) [58].

### 4.2 Lateral Cephalometry

following a systematic review, CNNs show high accuracy of landmark detection in 2D and 3D lateral cephalometry. The most frequent architectures were VGG‐19, YOLO V3, ResNet50, and ResNet34. Although the included studies showed a high risk of bias [53].

### 4.3 Panoramic Radiography

Recently, AI has facilitated the detection and segmentation of teeth in panoramic radiographs. Sensitivity and precision were reported at 98.9% and 99.6% respectively [52]. Panoramic films can provide data about growth assessment and chronological age. There are two reliable methods for dental age estimation, the D method and the W method. Based on seven left mandibular permanent tooth bud formation and development staging, chronological age will be estimated. Although the W method showed less overestimation compared to another one [60].

### 4.4 CBCT

Due to the high radiation dose, CBCT should not be used as routine radiography for diagnosis and treatment planning in orthodontics unless there is a persuasive reason. Complicated tooth impaction, root resorption, trauma, lip or palate cleft, dental or facial congenital anomalies, and temporomandibular joint abnormalities are convincing conditions for ordering CBCT [61]. The most challenging part of CBCT is the segmentation. Wang et al. designed a multiclass CNN called MS‐D networks (mixed‐scale dens) for jaw and teeth segmentation in CBCT. It diminished demanded time from 5 h (manual segmentation) to 25 s (AI) and performed accurately [62].

### 4.5 Photography

Among imagings in orthodontics, photography is noninvasive and radiation‐free for detailed intra and extra‐oral evaluation. One of the main goals of orthodontic treatment is facial appearance improvement by establishing facial proportions as “ideal” as possible [63, 64]. AI models speed up photography evaluation or orthodontic purposes including landmark detection, crowding categorization, and extraction decision. Different AI architecture models were used such as ResNet50, ResNet101, VGG16, and VGG19. VGGNet performed better than ResNet and the accuracy was higher in the maxilla compared to the mandible [64].

### 4.6 Growth Prediction

Craniofacial growth prediction plays an important role in the management of skeletal deformities and malocclusions. Forward growth of the mandible can be favorable and non‐favorable in Cl II and Cl III growth patterns, respectively [65]. Therefore, prediction of growth is notable during orthodontic treatments. Various methods have been available including hand‐wrist radiographs, cervical vertebrae maturation (CVM), and mandible growth prediction that have been combined with AI [47, 65]. The most important indicators of the evaluation of each method are written below:

a. Hand‐wrist radiograph: Third finger proximal/middle/distal phalanx, Fifth finger middle phalanx, Adductor sesamoid of thumb, Radius [66].
b. Cervical vertebrae maturation: inferior border and morphology of C2, C3, C4 [4].
c. Mandible growth: Mandibular Length and Y Axis (Sella to Gnathion) [65].

### 4.7 Skeletal Age Estimation: Reproducibility and Efficiency

Age estimation is applied for medical and forensic purposes based on dental and skeletal maturation [67, 68]. As we mentioned before, there are three main methods for measurement of skeletal age including, hand‐wrist radiography, CVM, and mandible growth [4, 65, 67]. Hand‐wrist radiography has been used for decades as a gold standard but due to some drawbacks, lateral cephalometry was replaced [67]. But the Reproducibility and Efficiency of CVM were still a question. A systematic review claimed that CVM might be an alternative to hand‐wrist radiography but further studies are required to have a definite decision [69].

With the entrance of technology in the field of orthodontics, CVM has been developed with artificial intelligence. But there were concerns about the reliability and accuracy of AI in chronological age estimation based on CVM. Studies have shown the accuracy of AI ranges from 50% to 90% [19]. Among the architecture of AI, the most accurate ones were ResNet152, DenseNet161, GoogLeNet, and VGG16 respectively [70].

### 4.8 Extraction or Non‐Extraction Therapy in Orthodontic Treatment

Extraction therapy is an approach in orthodontics that aims to relieve crowding and correct anteroposterior relationship in dental arches. Therefore, it provides demanded space for the treatment of discrepancies [35]. Many factors affect the decision of extraction in orthodontics including crowding, malocclusion, gender, age, soft tissue, etc [71]. A comprehensive evaluation is needed for making such an important decision by high‐experienced orthodontists [35].

Recently, decision‐making systems have been pioneered in orthodontic treatment planning. Computational modeling was used with a success rate of 90.4% in the extraction or non‐extraction approach [72]. One study designed a computer‐based decision‐making system for extraction and space prediction. Casts together with hard and soft tissue analysis in cephalometry were used as input indices (Table 1). However, two main factors should be considered the most: anterior teeth uncovered by incompetent lips and incisor mandibular plane angle (IMPA) [35].

**Table 1:** Input index in computational decision‐making system for extraction.

**Quantifiable indices**	
Cast measurement	Overbite Overjet Space for correcting Spee's curve Crowding in the upper and lower dental arches
Hard tissue cephalometriy	ANB Wits (mm) FMA (FH‐MP) FMIA(L1‐FH) IMPA (L1‐MP) L1‐NB L1‐NB (mm) U1‐SN U1‐NA U1‐NA (mm) U1‐L1
Soft tissue cephalometriy	NLA (Cm‐Sn‐UL) Ns‐Sn‐Pos UL‐Eplane (mm) LL‐Eplane (mm) Z angle
**Non‐quantification indices**	
Others	Situation of heredity Protruded anterior teeth uncovered by incompetent lips

Finally, the decision for tooth extraction before orthognathic surgery is notably challenging. due to the irreversible intervention of tooth extraction, it needs an accurate outcome which might be fulfilled with various studies with sufficient data in the future [73].

### 4.9 Orthognathic Surgery

The purpose of orthognathic surgery is to re‐establish oral function and esthetic [74]. Due to the complexity of maxillofacial structures, orthognathic surgery has always faced challenges [74]. Challenges can be categorized into four main issues: (1) evaluation of hard and soft tissues (2) subtle diagnosis, (3) an appropriate treatment plan, and (4) outcome prediction [74, 75]. With the entrance of artificial intelligence in orthodontics, mentioned challenges have been resolved to some extent.

### 4.10 Hard and Soft Tissues Evaluations

The definite decision about the need for orthognathic surgery is conceivable only after the evaluation of anatomical structures which can be done with an improved accuracy using AI. AI enhances the evaluation of hard and soft tissues in both radiography and facial photography within orthognathic surgery [76, 77]. Most studies use AI in craniomaxillofacial segmentation in cephalograms, CT, and CBCT for the identification of soft tissues (pharyngeal airway, lip, chin, etc.) and hard tissues (teeth, mandible, maxilla, cervical vertebrae) [76, 77, 78]. But still, there is a need for more data in CBCT, CT scans, and photogrammetry to integrate images [74]. Among AI frameworks, nnU‐Net seems to be beneficial in full automatic CT scan segmentation, and it is recommended for achieving an integrated system for the other imaging [79].

### 4.11 Diagnosis

After determining landmarks, craniofacial deformities will be diagnosed. The determination of the patient's developmental stage for a decision on surgery is important and it is indicated by hand‐wrist maturation (HWM) [74]. Hand‐wrist radiographs have been routinely used in orthodontics for many years to determine the peak of growth spurt. However, concerns have been raised about the extra radiation exposure from an additional radiograph. With the introduction of the CVM method for assessing skeletal age on a lateral cephalogram, it is now debatable whether an additional hand‐wrist radiograph is necessary for skeletal age evaluation. Reflecting this concern, the British Orthodontic Society's 2008 guidelines stated that hand‐wrist radiographs are no longer recommended for predicting the onset of the pubertal growth spurt [80]. cervical vertebrae maturation (CVM) is known as a reliable method for growth determination [69]. Determination of each developmental stage in CVM is hard to distinguish even for experts; however, AI is known as a high‐performance tool for this purpose [74].

### 4.12 Treatment Planning

A well‐designed treatment plan may lead to a successful outcome. It needs cephalometric analysis, study casts, and models. Recently treatment plans, are designed based on machine learning algorithms with acceptable results [81]. 3D shape models can guide the surgeon with the desired location of bones. SDNet architecture, using several encoding layers, is designed for bone correction in facial deformities [82]. But it should be noted that a single trained model is not enough for all possible deformities [81].

Orthognathic surgery is a multidisciplinary approach involving such specialists as orthodontists and surgeons. Treatment planning must be verbalized and visualized among practitioners with different expertise. A virtual setup seems to be beneficial in sharing therapeutic options and visualizing each intervention for the other practitioners to get familiar with different protocols [83].

### 4.13 Treatment Outcome Prediction

Nowadays, the prediction of future results is facilitated by employing AI algorithms. It is expected that orthognathic surgery establishes function and esthetic through occlusion adjustment and fixing facial deformities. According to recent studies, AI Predicted normal jaw shape and landmarks, facial symmetry, bone displacement, and soft tissue profile with 3D imaging and cephalometric analysis [82, 84]. 2D surgical planning is limited especially in patients with asymmetry [85]. 3D surgical planning in soft tissue prediction is acceptable among surgeons and patients. It guides surgeons during operations, increases patients' confidence and motivation, and diminishes preoperative anxiety [85, 86]. But patients should be aware of the variation between actual results and prediction; for example, results were more accurate in Cl III malocclusion and the discrepancy was less than 2 mm but lips and chin still showed the most discrepancy among other regions [86]. In the following studies, ten landmarks along with gender and age were evaluated for soft tissue postoperative prediction, however, further studies should consider fat deposit, muscle tone, skin type, and even ethnicity [86].

## 5 Estimation of Cervical Vertebrae Maturation Stages Using AI

Radiographs of hand wrist have been used as a reliable scale for determining skeletal development; however, due to additional x‐ray exposure, time, and cost, lateral cephalograms are considered as more available options which are often required before orthodontic treatment planning [9, 38, 87, 88]. chronological age is not a valid approach for estimation growth spurt time in patients. CVM stages is one of the suitable methods for assessing growth peak. This method is based on the specific morphological changes of cervical vertebrae [89, 90].

A recent study examining the staging of CVM by clinicians showed a relatively low reliability [67]. The intra‐rater agreement which was assessed by six experts ranged from 77.0% to 87.3%. The inter‐rater agreement was calculated 42.8% which is even worse than the previous one. The study also revealed the poor reproducibility for stage 3 assessment, a critical stage for growth peak determination. These findings are consistent with similar articles that indicate disagreement in CVM assessment by clinicians [14, 91, 92]. Conversely, a promising result of studies show 90% accuracy in CVM stage assessment by utilizing AI algorithms. AI will simplify decision‐making process and saves time in performing problematic tasks [7, 93].

AI algorithms perform tasks including in diagnosis, segmenting and categorizing cephalometric radiographs [94, 95]. AI algorithms extract features automatically and guide clinicians in complex data extractions with the least amount of knowledge and error. Despite several advantages of utilizing AI algorithms in CVM stage assessment, it has some issues including the high computational load of training AI models while medical imaging datasets have limited capacity, challenges in labeling data properly, gathering balanced data and difficulty in getting access of patients' radiographs due to moral issues [96, 97]. According to Tajmir et al. [17], AI enhances ability of radiologists to accurately and consistently determine the bone age of individuals. Additionally, it has been shown that using AI by a radiologist is more accurate than relying just on AI. The conventional methods need manual input of landmarks at various stages of the processing phase. The reliance on manual operation may lead to subjective decisions and lower reproducibility [98]. Previous studies that used traditional ML algorithms had limitationas, and these semi‐automated models required specialistis to manually extract characteristics and identify cervical vertebrae anatomical landmarks [18, 26, 99]. CNN algorithm proposed by Li et al. [70] required minimal data input and could reduce the workload for orthodontists as well as enhancing the accuracy of final results. This CNN model employed predictions based on the characteristics of C3 and C4 vertebrae which were comparable to those features used by orthodontists, but several minor details, such as the concavity in the inferior border of the C2 vertebra, were overlooked [7].

Recognizing the shape and contour of cervical vertebrae was the first step for CVM staging using automated‐AI models which is called labeling. Results of previous studies showed that AI labeling was matched with manual labeling as a gold standard method with a very low mean error of 0.36 mm. However, the mean error between two manual labelings was 0.48 mm. Therefore, AI labeling can be more reliable than manual labeling [100, 101]. A study revealed that ANN models have an accuracy of 77.02% in determination of CVM stages [102]. Another study by Makaremi et al. [38] showed a good performance of AI base models for CVM assessment. Therefore, due to the results of similar studies, automated‐AI base models can be considered as a more efficient and feasible methods for determination of CVM in orthodontics [103].

Neural networks (NNs) are deep‐learning based algorithms that has been successfully designed to determine CVM stages on lateral cephalograms, with over 90% accuracy of validation [19]. The Convolutional Neural Network (CNN) is the most promising and widely used trainable model utilized for classifying images into a certain number of classes [104]. Fully automated CNN model with the directional filters (CNNDF) proposed by Atici et al. achieved a validation accuracy of 84.63% in CVM stage classification into five classes. This model showed better performance than pre‐trained network models without the directional filters. Another underlying cause for the high accuracy of CVM classification reported for the model was the high number of images used as a data set [105]. Another study showed that the addition of two more steps to the model for region of interest (ROI) detection and segmentation would effectively restrict the model's focus exclusively to the cervical vertebrae, hence resulting in higher accuracy [106].

Summary of studies using AI models for estimation of cervical vertebrae maturation stages is available in Table 2.

**Table 2:** Summary of data extracted from studies using AI models for estimation of cervical vertebrae maturation stages.

Author, year	Data source	Sample size and structure	Age ranges of samples	Data processing models	Model architecture	Results	Conclusion
Makaremi et al. [38] (2019)	Not mentioned	1870 lateral cephalometric radiographs	Not mentioned	CNN model	Customized DL network	**Accuracy:** No more than 90%, increased when trained the model with more images	The proposed model and method are validated by cross‐validation and are ready for clinical use
Kok et al. [102] (2019)	Archive of Necmettin Erbakan University	300 lateral cephalometric radiographs	8–17 years	k‐nearest neighbors (k‐NN) Naive Bayes (NB) Decision tree (Tree) Artificial neural networks (ANN) Support vector machine (SVM) Random forest (RF) logistic regression (Log. Regr.)		**Classification accuracy:** Decision tree CSV1 (97.1%) > > ANN CSV1 (93%) > Decision tree CSV2 (90.5%) > ANN CSV2 (89.7%) > kNN CVS 6 (78.7%) > SVM CVS3 (73.2%) > kNN CVS5 (60.9%) > > SVM CVS4 (58.5%) > ANN CSV4 (55.6%) > kNN CVS2 (52.8%) > kNN CVS3 (50.9%) **The mean rank value:** ANN was the most stable algorithm with its 2.17 average rank	Decision tree and kNN were the algorithms with the highest accuracy in determining cervical vertebrae stages. The ANN algorithm was observed to have the second‐highest accuracy values and observed with most stable classification results
Amasya et al. [26] (2020)	Archive of Suleyman Demirel University	647 lateral cephalometric radiographs	10–30 years	Logistic regression (LR) Support vector machine Random forest Artificial neural network (ANN) Decision tree (DT)	CVM stage classifier: ANN Cervical vertebrae morphology classifiers: 1) concavity: LR 2) vertebral body shape: DT	**weighted kappa coefficient (K)** Concordance of AI models and expert evaluations ANN model: K = 0.926 LR model: K = 0.968 DT model: K = 0.949 **CVM staging** ANN > DT > RF > SVM > LR **Presence of concavity** LR > SVM > ANN > DT > RF**Vertebrae body shape** DT > RF > ANN > SVM > LR	ANN model had the best performance among 5 models
Amasya et al. [107] (2020)	archive of Suleyman Demirel University	647 lateral cephalometric radiographs	10–30 years	ANN model	ANN model	**weighted kappa coefficient (K)** wk = 0.92‐0.98	ANN model may replace human observers in CVM analysis
Kim et al. [106] (2021)	Jeonbuk National University Dental Hospital	600 lateral cephalometric radiographs	6–18	Model 1: Classifier model Model 2: ROI detector–classifier model Model 3: ROI detector–segmentation–classifier model	1. ROI detection: Attention U‐Net convolutional network 2. Segmentation: Attention U‐Net convolutional network	**ROI detection** IOU score: 0.9181 **Segmentation** Comparing ground truth and the output of the segmentor: 120 images were segmented into 3 regions 9 images were segmented with > 3 regions 3 test images were segmented with < 3 regions **CVM Classification:** Accuracies of the three models ε (0)Model 1: 0.5333 Model 2: 0.5916 Model 3: 0.6250 ε [1] Model 1: 0.9250 Model 2: 0.9667 Model 3: 0.9333	The three‐step segmentation‐based model produced the best accuracy (62.5%) compared to the models that were not segmentation‐based
Zhou et al. [101] (2021)	Archive of Sichuan University	1080 lateral cephalometric radiographs	6–22 years	CNN model	Detnet and resnet50 for Labeling and staging	**Training data set**: 980 **Test data set**: 100 **ICC**: 0.98 **Accuracy of staging**: 0.71**Precision:** CS2 = CS6 > CS4 > CS1 > CS5 > CS3 **Sensitivity:** CS5 > CS1 > CS6 > CS4 > CS3 > CS2 **Specificity:** CS2 = CS6 > CS4 > CS3 > CS1 > CS5**F1‐score** CS6 > CS1 > CS4 > CS5 > CS2 > CS1	Successful agreement of AI and experts in CVM
Mohammad‐Rahimi et al. [31] (2022)	Archive of Shahid Beheshti University of Medical Sciences Dental School	890 lateral cephalometric radiographs	C2 to C4 stages of cervical vertebra maturation	Transfer learning model	Customized ResNet‐101 model	**Six‐class CVM diagnosis: Model′s validation**: 62.63% **Test accuracy**: 61.62% **Three‐class CVM diagnosis: Model′s validation:** 75.76% **Test accuracy:** 82.83%	The newly developed AI model had reasonable accuracy in detecting the CVM stage and high reliability in detecting the pubertal stage; however, the accuracy was still less than that of human observers
Radwan et al. [108] (2023)	Archiveof Near East University	1501 lateral cephalometric radiographs	7–25 years	U‐Net Alex‐Net	Segmentation: U‐Net classification: Alex‐Net	**Segmentation Training data set:** 1201 images**Testing data set:** 150 images **Dice score:** 0.931 **Accuracy:** 0.998 **Weighted kappa coefficient (K):** 0.87 **ClassificationAccuracy** Pre‐pubertal > postpubertal > pubertal **Overall accuracy**: 0.802 **Sensitivity** postpubertal > pre‐pubertal> pubertal **Specificity** Pubertal> pre‐pubertal > post‐ pubertal **F1‐score** postpubertal > pre‐pubertal > pubertal	Successful in CVM segmentation and classification
Atici et al. [109] (2023)	American Association of Orthodontists Foundation (AAOF) Craniofacial Growth Legacy Collections	1018 lateral cephalometric radiographs	Not mentioned	CNN model	AggregateNet custom‐designed CNN model: a parallel structured deep CNN with a pre‐processing layer	**Performance:** custom‐designed CNN model (AggregateNet) > ResNet20, Xception, aMobileNetV2, and CNNDF	AggregateNet together with the tunable directional edge filters is observed to produce higher accuracy than the other models that we investigated in the fully automated determination of the CVM stages

### 5.1 Limitations

The review may be limited by the availability and accessibility of existing literature. There have been significant heterogeneity in the AI methodologies, datasets, and evaluation metrics used across different studies, making it challenging to compare results directly or synthesize them into a unified conclusion.

## 6 Conclusion

AI algorithms can be designed to successfully help the orthodontist in classifying CVM stages on lateral cephalograms. Several factors would affect the final results on accuracy validation rate such the size of the data set, the method used for landmark labeling (whether manual or automated), number and features of the designed layers, etc. The utilization of these models is recommended by an expert, and a fundamental understanding of the CVM staging approach is necessary to enhance the precision of skeletal age estimation. It is important to keep in mind that AI technology serves only as an assistant rather than a replacement for human expertise.

## Author Contributions

**Pegah Farzanegan:** conceptualization, writing – original draft, methodology. **Mobina Zarabadi:** conceptualization, writing – original draft, methodology. **Shaghayegh Najary:** conceptualization, writing – review and editing, methodology. **Mohammad Ali Tahmasbi:** conceptualization, methodology, writing – review and editing. **Mohammad Behnaz:** conceptualization, writing – review and editing, supervision.

## Ethics Statement

The authors have nothing to report.

## Conflicts of Interest

The authors declare no conflicts of interest.

## Transparency Statement

The lead author Mohammad Behnaz affirms that this manuscript is an honest, accurate, and transparent account of the study being reported; that no important aspects of the study have been omitted; and that any discrepancies from the study as planned (and, if relevant, registered) have been explained.

## Data Availability

The datasets used and/or analyzed during the current study available from the corresponding author on reasonable request.
